# Novel CD123 polyaptamer hydrogel edited by Cas9/sgRNA for AML-targeted therapy

**DOI:** 10.1080/10717544.2021.1934191

**Published:** 2021-06-12

**Authors:** Haibin Wu, Liyu Zhang, Zeen Zhu, Chenxi Ding, Shengquan Chen, Ruiping Liu, Huafeng Fan, Yang Chen, Hui Li

**Affiliations:** a Department of Neonatology, the First Affiliated Hospital of Xi’an Jiaotong University, Xi’an, Shaanxi, China; b Shaanxi Institute of Pediatric Diseases, Affiliated Children’s hospital of Xi’an Jiaotong University, Xi’an, China; c Department of Hepatobiliary Surgery, The First Affiliated Hospital of Xi'an Jiaotong University, Xi'an, China; d Department of Clinical Nutrition, Affiliated Children’s Hospital of Xi’an Jiaotong University, Xi’an, China; e Department of Cardiovascular Medicine, Affiliated Children’s Hospital of Xi’an Jiaotong University, Xi’an, China; f Department of Neonatology, Affiliated Children’s Hospital of Xi’an Jiaotong University, Xi’an, China

**Keywords:** CD123 aptamer, Cas9/sgRNA, controlled release, AML, polyaptamer hydrogel

## Abstract

CD123 targeting molecules have been widely applied in acute myelocytic leukemia (AML) therapeutics. Although antibodies have been more widely used as targeting molecules, aptamer have unique advantages for CD123 targeting therapy. In this study, we constructed an aptamer hydrogel termed as SSFH which could be precisely cut by Cas9/sgRNA for programmed SS30 release. To construct hydrogel, rolling-circle amplification (RCA) was used to generate hydrogel containing CD123 aptamer SS30 and sgRNA-targeting sequence. After incubation with Cas9/sgRNA, SSFH could lose its gel property and liberated the SS30 aptamer sequence, and released SS30 has been confirmed by gel electrophoresis. In addition, SS30 released from SSFH could inhibit cell proliferation and induce cell apoptosis *in vitro*. Moreover, SSFH could prolong survival rate and inhibit tumor growth via JAK2/STAT5 signaling pathway *in vivo*. Additionally, molecular imaging revealed SSFH co-injected with Cas9/sgRNA remained at the injection site longer than free aptamer. Furthermore, once the levels of cytokines were increasing, the complementary sequences of aptamers injection could neutralize SS30 and relieve side effect immediately. This study suggested that CD123 aptamer hydrogel SSFH and Cas9/sgRNA system has strong potential for CD123-positive AML anticancer therapy.

## Introduction

Acute myeloid leukemia (AML) is one of the most common types of leukemia derived from myeloid progenitor cells around the world, with an average 5-year survival of ∼28% (Ehx et al., 2021; Liu, 2021; Xuan & Liu, 2021). At present, the major treatment for AML involves induction chemotherapy and hematopoietic stem cell transplantation (HSCT), etc. (Carter et al., 2020; Appelbaum, 2021). However, despite constant emergence of these treatment options, relapse is still a common scenario and the most challenging aspect in AML of contemporary oncology. About one-third and one-half of patients will relapse after a transient remission, with a median survival of less than 6 months and estimated 5-year disease-free survival (DFS) of 20–40% (Pasquer et al., 2021). The most major reason for AML relapse is residue of AML cells. Current chemotherapeutic agents are unable to distinguish tumors from normal tissues (Wu et al., 2017), resulting in damaging CD123-positive normal cells as well. These features may generate serious problematic side effects, including limited drug intensity, duration of chemotherapy and reduced therapeutic efficacy, resulting in treatment failure and relapse (Liu, 2021; Rowe, 2021).

Targeted therapy, one of the most effective methods for cancer treatment, is a promising method for AML treatment. Targeted therapy could deliver drugs and inhibit AML cells selectively but not to their normal counterparts (Stuani et al., 2021). The alpha chain of interleukin 3 receptor (IL3R-α), designated as CD123, has been validated a mainstream target for AML. CD123 is highly expressed on several hematologic neoplasms, including AML, acute lymphoblastic leukemia (ALL), blastic plasmacytoid dendritic cell neoplasm (BPDCN), hairy cell leukemia and certain lymphomas, but expressed at a low level or to be absent on normal hematopoietic stem cells. Further, CD123 is also highly expressed on leukemic stem cells (LSCs), the origin of AML cells. Interestingly, patients with high CD123 expression blasts showed poor outcome and higher relapse rate. In addition, CD123 is a significant factor to control proliferation, growth, and differentiation of AML cell via the activation of many signal pathways such as JAK/STAT (Shi et al., 2019; Bulaeva et al., 2020; Lane, 2020; Sugita & Guzman, 2020). At present, CD123 targeting therapy for AML are in advanced preclinical and clinical development, and they exhibit robust anti-leukemic activity, including antibody–drug conjugate (ADC), bispecific T-cell engager (BiTE) and chimeric antigen receptor T-Cell immunotherapy (CART) (Gill, 2019; Slade and Uy, 2020). However, some advantages limited the clinical application of these therapeutics: (1) fatal side effects: CD123 is expressed at a low level in some epithelial cells and monocytes may cause side effects such as cytokine storm, capillary infiltration syndrome, hepatic transaminase elevation, hypoalbuminemia and myelosuppressi (Cartellieri et al., 2016; Sun et al., 2019); (2) resistance: the immune system of some patients may be provoked by antibodies and generate several adverse effects (Togami et al., 2019); (3) complex design and high cost: since sensitive to temperature, pH and multigelation, antibodies are easier to lose their functions, and these drugs are laboriously prepared, limited drug loaded and highly cost. Therefore, novel CD123-based AML targeted therapy was urgently needed.

Aptamer, another kind of targeted molecules, has presented as a powerful clinical potential tool for AML targeted therapy (Zhu & Chen, 2018). Aptamers are composed of single-stranded oligonucleotides (DNA or RNA), which could form a specific three-dimensional (3D) structure to recognize its targets with high specificity and affinity (Wan et al., 2019). When compared with antibodies, aptamers have obvious advantages: high specificity and affinity to targets, easy synthesis, limited pharmaceutical cost, multiple modification strategy, no immunogenic properties, easy penetration to tumor tissues, and easy storage and transportation (Sun & Zu, 2015). Recent works of Dianping Tang et al. have fully demonstrated the merit of aptamer in bio-applications (Qiu et al., 2019; Zeng et al., 2019; Lv et al., 2020; Lu et al., 2021). To date, a plenty of aptamers have been generated and have showed great potential in pre-clinic. In our previous study, we generated the first ever CD123 thioaptamer, termed as SS30, which could bind CD123 with high specificity and affinity (Hu et al., 2019; Zhao et al., 2021). SS30 could impair the function of CD123 molecule by competing with IL-3 to bind to CD123, and could down-regulate the expression of *p-AKT* and *p-STAT5*, resulting in inhibitory effect on CD123^+^ AML tumor cells *in vitro* and *in vivo*. Our data have fully validated that SS30 is a novel anti-tumor agent with therapeutic potential for AML. However, since the small size, they do not show strong retention in the body. To increase the retention of SS30 in the body, DNA hydrogel, a kind of important DNA materials was chosen. DNA hydrogel could retain the biological function of DNA, and realizes the perfect fusion of structure and function of hydrogel materials, presenting good biocompatibility, adjustable biodegradability and controllable mechanical properties (Li et al., 2019; Zhang et al., 2020). Moreover, to precisely cleave the DNA hydrogel to generate the precise aptamer for further inhibition function, CRISPR-associated protein 9 (Cas9) was utilized. Cas9 has been widely used in the context of gene editing, such as in therapeutics and agricultural products (Lee et al., 2018). Jaiwoo Lee et al. have successfully designed an (rolling circle amplification) RCA-based DNA hydrogel that can release PD-1 aptamers via Cas9/sgRNA-mediated specific editing (Lee et al., 2019). This hydrogel not only exhibits prolonged retention at tumor sites *in vivo* but also inhibits the activity of immune cells in the tumor microenvironment.

Here in this study, we first generate a DNA hydrogel composed of SS30 using RCA method, termed as SS30 polyaptamer hydrogel (SSFH), and Cas9/sgRNA was used to release SS30 from SSFH in a sustained manner at the site of administration. It was demonstrated that this DNA hydrogel could prolong the circulation time and retention of SS30, and enhanced SS30 concentration in tumor tissues. In addition, this DNA hydrogel had a marked inhibitory effect on CD123^+^ AML tumor cells *in vitro* and *in vivo*. Furthermore, this DNA hydrogel produced a prolonged survival of model animals in vivo. Most importantly, once the generated cytokines increasing, the complementary sequences of aptamers injection could relieve immediately. Overall, the present *in vitro* and *in vitro* data as well as mechanistic studies fully validate that DNA hydrogel made of SS30 is a novel anti-tumor agent with therapeutic potential for AML.

## Materials and methods

### Reagents

The RCA template and primers were synthesized by Sangon Biotech (Shanghai, China). T4 DNA ligase kit was purchased from Sangon Biotech (Shanghai, China). phi29 DNA polymerase (100 units/mL) was purchased from Thermo Scientific (Waltham, MA). RPMI-1640 medium was obtained from Hyclone (Thermo Scientific, Waltham, MA). Fetal bovine serum was purchased from Gibco (Invitrogen, Carlsbad, CA). GeneArt precision gRNA synthesis kit as obtained from Thermo Scientific (Waltham, MA). Cas9 protein was purchased from PNA BIO INC (Newbury Park, CA). RNase (0.25 mg/ml) was obtained from Qiagen (Valencia, CA). CCK8 assay kit (ab228554) was purchased from Abcam (Cambridge, UK). CellTiter 96® AQueous One Solution Cell Proliferation Assay (MTS) was obtained from Promega (Madison, WI). BrdU Cell Proliferation ELISA Kit (colorimetric) (ab126556) was purchased from Abcam (Cambridge, UK).

### Cell lines and culture

The human B-cell precursor leukemia cell line RCH-ACV was obtained from Cell Culture Center of Peking Union Medical College (Beijing, China). The human acute myelocytic leukemia cell line Molm-13 was purchased from ATCC (Manassas, VA). Cells were cultured in RPMI-1640 medium, supplemented with 10% fetal bovine serum (FBS, Gibco, Carlsbad, CA) and a mixture of penicillin/streptomycin. Additionally, 5 ng/ml IL-3 was also added into cell culture buffer. Cells were cultured at 37 °C in a humidified atmosphere with 5% CO_2_. All experiments were performed on cells in the exponential growth phase.

### Construction of SSFH

To generate SS30 formed hydrogel (SSFH), first, rolling circle amplification (RCA) template should be designed, which could generate CD123 aptamers SS30 by Cas9/sgRNA-specific cleavage. Each template contained SS30 aptamer sequence and sgRNA target sequence. The sequences were as follows: template 1, 5′-CCGCCCAAATCCCTAAGAGGCAGGGAGTTCGCTAGTAGCTACGGGACCAGACACAGTACACACGCA**CCCTGAAGTTCATCTGCACCACC**-3′; template 2, 5′-**AACTTCA**CCGCCCAAATCCCTAAGAGGCAGGGAGTTCGCTAGTAGCTACGGGACCAGACACAGTACACACGCA**GGTGGTGCAGATG**-3′, where the template for the SS30 is underlined and that for the sgRNA target sequence is bold. Before the RCA process, pre-circular RCA template should be generated. 0.5 mM of each DNA template (Sangon Biotech, Beijing, China) was mixed with primer in hybridization buffer (1 mM EDTA, 10 mM Tris HCl, 100 mM NaCl, pH 8.0). The primer for template 1 was 5′-GTCGCTCGGTGGTGCAGATGAA-3′, The primer for template 2 was 5′-TCCCCTGAAGTTCATCTGCACC-3′. After the hybridization, the mixture was reacted with T4 DNA ligase (Sangon Biotech, Beijing, China) in T4 DNA ligase buffer (500 mM Tris-HCI；100 mM MgCl_2_；50 mM DTT；10 mM ATP；pH 7.6 @ 25 °C) overnight at 16 °C to close the nick. To inactivate T4 DNA ligase, the product was heated at 70 °C for 10 min. The SSFH was synthesized as followed: pre-circular RCA template was mixed with 50 mM Tris-HCl pH 7.5, 10 mM MgCl_2_, 10 mM (NH_4_)_2_SO_4_, 4 mM DTT, and phi29 DNA polymerase (100 units/ml; Thermo Scientific, Waltham, MA) for 48 h at 42 °C, and phi29 DNA polymerase was inactivated at 10 min at 65 °C. All the products were centrifuged 12,000 rpm for 15 min, and precipitate were re-suspended in triple distilled water (TDW). Template 1 and 2 were mixed at a 1:1 wt ratio for further study.

### The precise cleavage of SSFH by Cas9/sgRNA

gRNA was synthesized via a GeneArt precision gRNA synthesis kit (Thermo Scientific). The sgRNA sequence was: 5′-GGUGGUGCAGAUGAACUUCAGUUU UAGAGCUAGAAAUAGCAAGUUAAAAUAAGGCUAGUCCGUUAUCAACUUGAAAAAGUGGCACCGAGUCGGUGCUUUU-3′ (the target sequence is underlined). Then, to evaluate whether Cas9/sgRNA complex were assembled, first, Cas9 protein was fixed on COOH-modified magnetics beads via NHS/EDC reaction. In brief, 6 × 10^5^ carboxylated magnetic beads were washed by 200 μl MES (100 mM, pH 5.0) at room temperature twice. Then, beads were activated by 100 μl 1-ethyl-3-(3-dimethyllaminopropyl)-carbodiimide hydrochloride (EDC) (20 mg/ml) and 100 μl N-hydroxysuccinimide (NHS) (20 mg/ml) for 15 min with gentle stirring. Next, beads were washed by linking buffer (5.3 ml 0.2 M sodium dihydrogen phosphate and 94.7 mL 0.2 M sodium hydrogen phosphate). About 5 μg Cas9 protein (5 mg/ml, PNA BIO INC, Newbury Park, CA) was added to the beads and incubated at room temperature for 2 h. At last, beads were washed three times with PBS buffer and incubated with FAM-sgRNA (sgRNA chemically modified with FAM). Flow cytometry was applied to assess fluorescent signals. Beads coated with bovine serum albumin (BSA) and blank beads were treated as negative controls. Further, to evaluate the formation of SSFH/Cas9/sgRNA, Cas9 was fixed on beads and sgRNA was added to form Cas9/sgRNA. Then, Cas9/sgRNA beads incubated with FAM-labeled SSFH and applied for flow cytometry. Blank beads were treated as negative control. The zeta of Cas9/sgRNA was evaluated by particle size analyzer.

To induce Cas9-mediated cleavage, first, to evaluate the diameter change after Cas9 cleavage, SSFH (10 mM) was mixed with 6 × 10^5^ Cas9/sgRNA coated beads. After incubation, beads were removed under magnetic field and DNA mixture was assessed by particle size analyzer. Then, to further observe cleavage effect, Cas9/sgRNA complex were assembled. Cas9 protein (5 mg/ml, PNA BIO INC, Newbury Park, CA) was mixed sgRNA to form Cas9/sgRNA complexes. Then, different ratio of SSFH/Cas9/sgRNA complex was mixed and incubated at 37 °C for a range of time. The reaction mixtures were treated with RNase (0.25 mg/ml; Qiagen, Valencia, CA) to eliminate the sgRNA. About 1% agarose gel electrophoresis was applied to assess the cleavage of mixtures. The band density was analyzed using gene tools software from Syngene (Frederick, MD).

### Assessment of swelling rate

To calculate the swelling ratio, SSFH was freeze dried first. The freeze-drying powder of SSFH was mixed with triple-distilled water with or without Cas9/sgRNA. The weights were calculated at various time points, ranging from 1 h to 10 h. The swelling rate was calculated by this equation: swelling rate (%) = [(W1 − W2)/W1] × 100%. The W1 represented the weight of hydrated gel, whereas W2 is the weight of SSFH freeze drying powder.

### Binding ability evaluation

RCA template was subjected to PCR amplification. To evaluate binding ability, primers were labeled with FAM.

By flow cytometry: 1 × 10^5^ cells (Molm-13 and RCH-ACV) were incubated with 50 nM SS30, SSFH or cleavage mixtures at 37 °C for 30 min. Cells were washed by PBS and analyzed by flow cytometry. The mean fluorescence intensities (MFI) of FAM were analyzed.

By confocal microscope: 1 × 10^5^ cells (Molm-13 and RCH-ACV) were incubated with 5 μM SS30, SSFH or cleavage mixtures at 37 °C for 30 min. Cells were washed by PBS and analyzed by confocal microscope.

### Competing assay

1 × 10^5^ cells (Molm-13) were incubated at 96-well plate and washed by PBS twice. 50 nM FAM-labeled CD123 antibody and increasing concentrations of free SS30 or SSFH/Cas9 were incubated with cells at 37 °C for 30 min. Cells were centrifuged, supernatant fluid were collected and analyzed by fluorometer. The mean fluorescence intensities (MFI) of FAM were analyzed.

### Evaluation of anti-cancer ability in vitro

*Cell viability evaluation by CCK8 assay*: 1 × 10^5^ cells (Molm-13) were collected and seeded in 96-well plate. Cells were washed by PBS twice to remove FBS. Cells were treated with PBS, SS30 (20 mM), SSFH (20 mM), SSFH/Cas9/sgRNA(20 mM), or random library (5′-TGCGTGTGTAGTGTGTCTG-(N_28_)-CTCTTAGGGATTTGGGCGG-3′, 20 mM) for at 37 °C for 6 h. Then cells were washed by PBS and cultured for a further 48 h. The supernatant fluid was collected the cell proliferation were detected by CCK8 kit according to the manufacturer's standard protocol. The statistical difference was compared to the PBS group (* indicated *p* < .05; ** indicated *p* < .01).

*Cell viability evaluation by MTS assay*: 1 × 10^5^ cells (Molm-13) were collected and seeded in 96-well plate. Cells were washed by PBS twice to remove FBS. Cells were treated with PBS, SS30 (20 mM), SSFH (20 mM), SSFH/Cas9/sgRNA(20 mM), or random library(20 mM) for at 37 °C for 6 h. Then cells were washed by PBS and cultured for a further 48 h. MTS buffer was added in each well and further incubated for 2–4 h. The absorbance value was detected 490 nM. The statistical difference was compared to the PBS group (* indicated *p* < .05; ** indicated *p* < .01).

*Cell proliferation evaluation by BrdU assay*: 1 × 10^5^ cells (Molm-13) were collected and seeded in 96-well plate. Cells were washed by PBS twice to remove FBS. Cells were treated with PBS, SS30 (20 mM), SSFH (20 mM), SSFH/Cas9/sgRNA(20 mM), or random library(20 mM) for at 37 °C for 6 h. Then cells were washed by PBS and cultured for a further 48 h. About 100 μl supernatant fluid was added to reaction plate at 37 °C for 120 min. Then, plate was washed by washing buffer and 100 μl the first antibody buffer was added and incubated at 37 °C for 60 min. Next, plate was washed by washing buffer and 100 μl substrate working buffer was added at 37 °C for 15 min. About 100 μl terminating fluid was mixed and the absorbance value was detected at 450 nM. The statistical difference was compared to the PBS group (* indicated *p* < .05; ** indicated *p* < .01).

### Apoptosis and cell death analysis of AML cell lines

About 1 × 10^6^ cells (Molm-13) were collected and seeded in 6-well plate. Cells were washed by PBS twice to remove FBS. Cells were treated with PBS, SS30 (20 mM), SSFH (20 mM), SSFH/Cas9/sgRNA (20 mM), or random library (20 mM) for at 37 °C for 6 h. Then cells were washed by PBS and cultured for a further 48 h. Cells were incubated with TUNEL buffer and assessed according to the manufacturer's standard protocol.

### Assessment of retention ability of SSFH in vivo

The protocol of the animal study in this paper was reviewed and approved by the Ethics Committee of Xi'an Jiaotong University Affiliated Children's Hospital (Xi'an Children's Hospital, Xi'an, China), no. C2018004. Eight-week-old female BALB/c mice were purchased from the Xi'an JiaoTong University Lab Animal Center (Xi'an) and raised under pathogen-free conditions. 2 × 10^7^ *in vitro*-propagated Molm-13 cells were injected into both flank of BALB/c mice. FAM-labeled SS30 (5 μM), SSFH (5 μM), and SSFH/Cas9/sgRNA (5 μM) were injected into the hind legs of BALB/c mice. The retention of each sample at the injection site was observed by monitoring the FAM signal using an IVIS® Spectrum CT (PerkinElmer, Waltham, MA).

### Evaluation of anti-cancer effects of SSFH/Cas9 in vivo

To assess whether SSFH/Cas9 could inhibit AML proliferation and prolong retention *in vivo*, 2 × 10^7^ *in vitro*-propagated Molm-13 cells were injected subcutaneously into the flank of BALB/c mice to generate the mouse xenograft tumor model. Fourteen days later, treatments were initiated. The mice were divided into four groups, with six in each group: ① treated with saline once a day; ② treated with SS30 every 3 days (2 mM/kg); ③ treated with SSFH every 3 days (2 mM/kg); ④treated with SSFH/Cas9/sgRNA once every 3 days (2 mM/kg). The agents were administered through tail vein injection. The tumor volume was calculated using the following equation: tumor volume = tumor length × tumor width^2^/2. The body weights and survival rate were assessed and calculated. Subsequently, when the tumor volume was over 2000 mm^3^, or the mice that lost over 15% of their pretreatment body weights, the mice were euthanized by broken neck. Tumor tissues were collected and subjected to JAK2/STAT5 staining to evaluate anti-cancer effects of SSFH.

### Cytokine storm rescue ability

The mouse xenograft tumor models were divided into three groups and injected with saline, excessive SSFH/Cas9 or CD123 antibody to induce cytokine storm, respectively. Seven days later, blood samples were collected and subjected to detect inflammatory cytokines (TNF-α, IL-1, IL-6, IL-12, IFN-α, IFN-β, IFN-γ, and IL-8). When the SSFH/Cas9/sgRNA group and the CD123 antibody group exhibited an obvious difference compared with the saline group, agent injections should be ended. Then, mice in the saline group and the CD123 antibody group were injected with saline and mice in the SSFH/Cas9/sgRNA group were injected with the complementary sequence of SS30 (5′-CCGCCCAAATCCCTAAGAGGCAGGGAGT TCGCTAGTAGCTACGG GACCAGACACAGTACACACGCA-3′). Three days later, blood samples of mice were collected and subjected to detect inflammatory cytokines.

### Statistical analysis

All statistical analyses were performed using the SPSS11.0 software (SPSS, Chicago, IL) applied from Xi'an Jiaotong University. All numerical data were expressed as the mean ± standard deviation. Differences between the groups were examined with Student's two tailed *t*-test or one-way ANOVA. ANOVA was performed to evaluate the difference followed by Tukey post hoc test. Kaplan–Meier analysis was used to analyze the overall survival. Independent sample *t*-test was used to analyze the variance of experimental design. *p*-values of < .05 were considered statistically significant. * indicated *p* < .05, ** indicated *p* < .01.

## Results

### Construction and application of SSFH hydrogel

**Figure 1.**
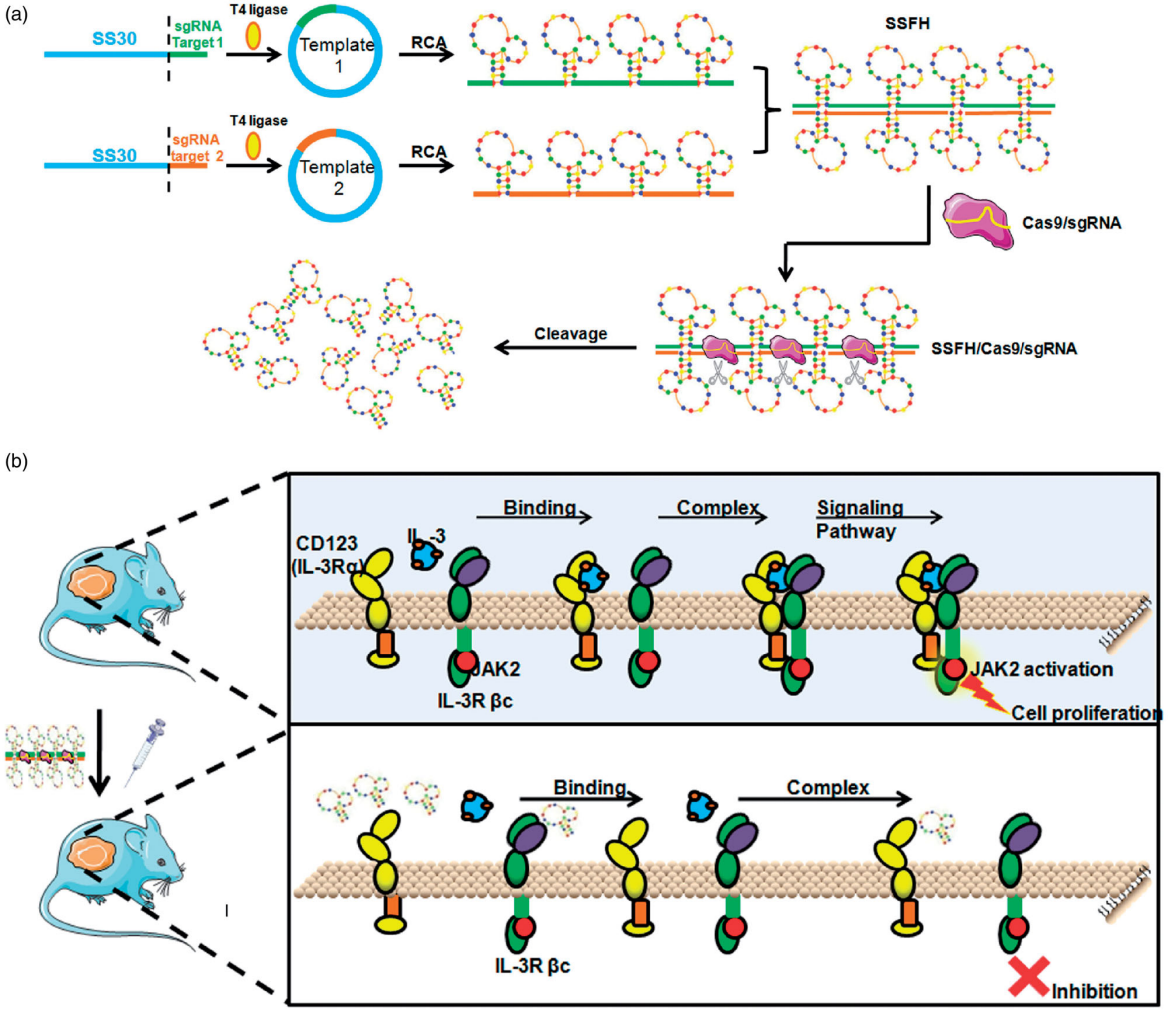
Illustration of novel CD123 polyaptamer hydrogel edited by Cas9/sgRNA for AML targeted therapy and its action mechanism. (a) To construct CD123 polyaptamer hydrogel edited by Cas9/sgRNA, two types of RCA templates were designed. Both contained the CD123 aptamer SS30, but they differed in that one contained the sgRNA target sequence for Cas9/sgRNA-specific cleavage, while the other contained the complementary sgRNA target sequence. The two RCA products were crosslinked by sequence-specific hybridization between the sgRNA target and complementary sequences. (b) In the tumor microenvironment, the precise excision of the CD123 aptamer by Cas9/sgRNA can release a plenty of SS30 and further block the interaction between IL-3 and CD123, interfering activation of JAK2/STAT5 signaling pathway.

To construct a Cas9-cleavable SS30 polyaptamer hydrogel (SSFH), as illustrated in Figure 1, first, two RCA templates were designed. RCA template 1 contained SS30 and sgRNA target sequence 1 and RCA template 2 contained SS30 and sgRNA target sequence 2. sgRNA target sequence 1 and sgRNA target sequence 2 were complementary sequences. In details, the composition of each RCA template is illustrated in Figure 1(A). One RCA template could be designed to generate long repeats of the SS30 aptamer and the sgRNA target sequence, and the other could be used generate long repeats of the SS30 aptamer and the corresponding sgRNA target-binding sequence. Therefore, after PCR amplification, two RCA products were hybridized between the sgRNA target, this could form a gel which was termed as SSFH. Additionally, since there generated a double-strand sgRNA target-binding sequence, once there was Cas9 presented, sgRNA could guide the enzyme in providing specific cleavage and cut SSFH into SS30. Furthermore, as presented in Figure 1(B), when SSFH and Cas9/sgRNA were both injected, Cas9/sgRNA cut SSFH and SS30 aptamer could compete with IL-3 to bind with CD123. When SS30 bond with CD123, it could interfere JAK2/STAT5 signaling pathway, and further inhibit cell proliferation (Hercus et al., 2014).

### Characterization of Cas9-cleavable SS30 polyaptamer hydrogel

**Figure 2.**
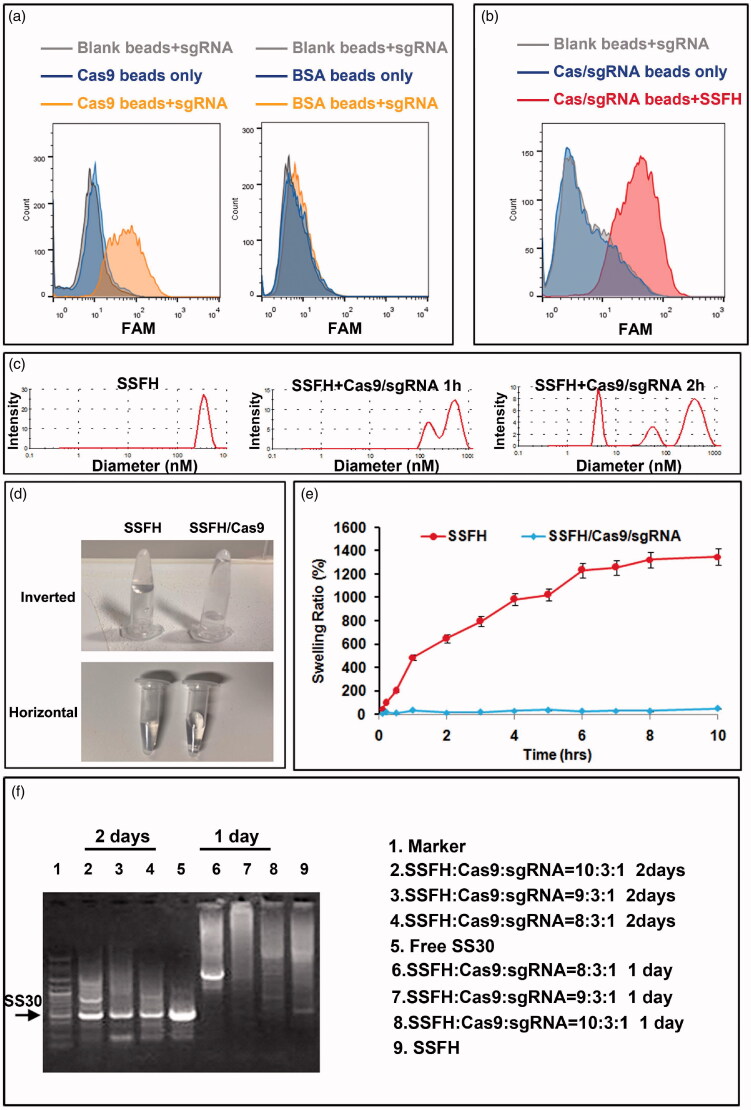
Physicochemical properties of the hydrogel. (a) Formation of Cas9/sgRNA assessed by flow cytometery. (b) Formation of SSFH/Cas9/sgRNA assessed by flow cytometery. (c) SSFH diameter size change after Cas9/sgRNA cleavage. (d) Morphologies of SSFH in the absence and presence of Cas9/sgRNA. Photographs show (left) SSFH before addition of Cas9/sgRNA, with the container inverted to show the gel property, and (right) SSFH/Cas9 incubated, with the container inverted to show the loss of gelation. (e) The swelling rate of Cas9/PAH was tested by soaking the lyophilized powder in triple-distilled water. (f) Cas9/sgRNA-edited cutting and release of the SS30 aptamer from SSFH. The cutting and release of the CD123 aptamer from SSFH was tested by gel electrophoresis. SS30, SSFH alone, or SSFH/Cas9 (incubated together for the indicated days) were electrophoresed on an agarose gel.

To assess whether SSFH was constructed and could be cleavable by Cas9 further, physicochemical behaviors were evaluated firstly. First, to evaluate whether Cas9/sgRNA complex was formed, Cas9 protein was fixed on beads and incubated with FAM-labeled sgRNA. Flow cytometry was applied to assess fluorescent signal. As presented in Figure 2(A), sgRNA could bind to Cas9 obviously but not to blank beads. Whereas, sgRNA did not bind with BSA beads. This data indicated a successful Cas9/sgRNA formation. The zeta potential was -5.33 mV. Additionally, to assess SSFH/Cas9/sgRNA complex, Cas9/sgRNA was fixed on beads and incubated with FAM-SSFH. As shown in Figure 2(B), there exhibited a stronger binding of SSFH with Cas9/sgRNA, but not in blank beads, indicating a formation of SSFH/Cas9/sgRNA complex. Further, to detect cleavage of SSFH mediated by Cas9, after incubation with Cas9/sgRNA, SSFH mixture was analyzed for diameter. As shown in Figure 2(C), before cleavage, the average diameter of SSFH was 567 nM. After incubation with Cas9/sgRNA for 1 h, a smaller size about 217 nm appeared. After incubation with Cas9/sgRNA for 2 h, the diameter of SSFH mixture was decreased significantly. These data suggested a successful cleavage of SSFH by Cas9. Furthermore, as presented in Figure 2(D), when in the absence of Cas9/sgRNA, SSFH exhibited an original position at the bottom of tube which was subjected to inversion, indicating gel characteristics. However, after the incubation with Cas9/sgRNA, the mixture flowed to the underside of the lid while the movement of inversion. Additionally, the swelling behavior of the hydrogel also differed in the absence and presence of Cas9/sgRNA. SSFH was freeze dried firstly. The freeze-drying powder of SSFH was mixed with triple-distilled water with or without Cas9/sgRNA. The weights were calculated at various time points. As shown in Figure 2(E), the lyophilized SSFH powder could rehydrate to hydrogel directly, and showing a swelling ratio of 659.47 ± 23.4% over 2 h. However, after incubation with Cas9, Cas9/SSFH did not exhibit such swelling function. Furthermore, to evaluate whether Cas9 could cleave SSFH into single SS30, different ratio of SSFH/Cas9/sgRNA complex was mixed and incubated at 37 °C for a range of time. The products were assessed by 1% agarose gel electrophoresis. As presented in Figure 2(F), there generated more SS30 for incubation 2 days than 1 day, and when the weight ratio of PAH, Cas9, and sgRNA was 8:3:1, and incubation time was over 2 days, SSFH could be cleaved into single SS30 completely (full liberation of the CD123 aptamer SS30).

### Binding ability evaluation

**Figure 3.**
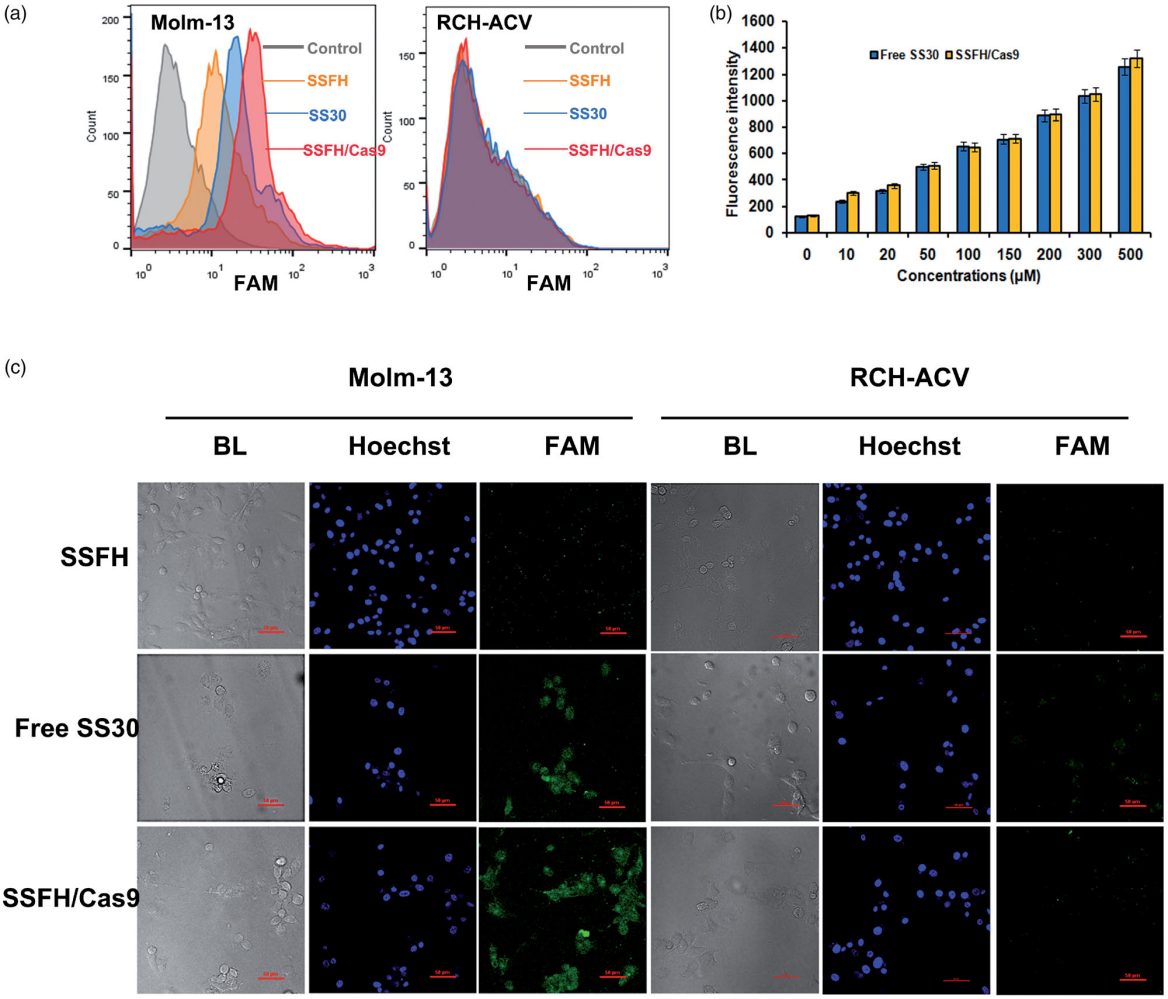
Evaluation of binding ability. (a) Flow cytometry assessment of SSFH/Cas9 for CD123 binding specificities. FAM-labeled DNA sequences were incubated with CD123-positive cells Molm-13 and CD123-negative cells RCH-ACV. Fluorescence signal was assessed. (b) Competing assay. Molm-13 cells were incubated with FAM-labeled CD123 antibody and increasing concentrations of free SS30 or SSFH/Cas9. Cells were centrifuged, supernatant fluid were collected and analyzed by fluorometer. The mean fluorescence intensities (MFI) of FAM were analyzed. (c) Confocal microscope assessment of SSFH/Cas9 for CD123 binding specificities.

To assess the binding ability of SSFH/Cas9/sgRNA 50 nM FAM-labeled SS30, SSFH or SSFH/Cas9/sgRNA cleavage mixtures were incubated with Molm-13 or RCH-ACV, respectively. Cells were observed by confocal microscope and fluorescence intensity was assessed by flow cytometry. As presented in Figure 3(A), when compared with SS30 and SSFH/Cas9/sgRNA, SSFH generated a relatively lower in CD123-positive Molm-13 cells. It may due to a restricted and un-exposed structure of SS30 in SSFH. Free SS30 or SSFH/Cas9/sgRNA both exhibited a strong binding trend to CD123-positive Molm-13 cells, indicating a successful release of SS30 in SSFH/Cas9/sgRNA, and still remained binding ability to CD123-positive cells. However, all these agents did not bind to CD123-negative RCH-ACV cells. This result was also consistent with confocal microscope. As shown in Figure 3(C), SSFH did not generate evident fluorescence intensity in Molm-13, whereas there showed a significant fluorescence signal in the free SS30 and the SSFH/Cas9/sgRNA group (*p* < .05). SS30, SSFH/Cas9/sgRNA, and SSFH did not show obvious signal in RCH-ACV cells. These results suggested that SSFH/Cas9/sgRNA still maintained CD123 binding ability after Cas9 cleave.

### Binding target confirmation

To validate the target of SSFH/Cas9/sgRNA was CD123 but no other proteins expressed on CD123-positive cells, competing assay was applied. In brief, Molm-13 cells were incubated with 50 nM FAM-labeled CD123 antibody first, and increasing concentrations of SSFH or SSFH/Cas9 was added. Fluorescence intensity of supernatant was detected and shown in Figure 3(B). With an increasing concentration of SSFH/Cas9, the FAM fluorescence intensity was increased, and as well as the free SS30 group, indicating a competing between CD123 antibody and SSFH/Cas9. These data suggested the target of SSFH/Cas9/sgRNA was still CD123.

### SSFH/Cas9 inhibits the proliferation of CD123-positive cells in vitro

**Figure 4.**
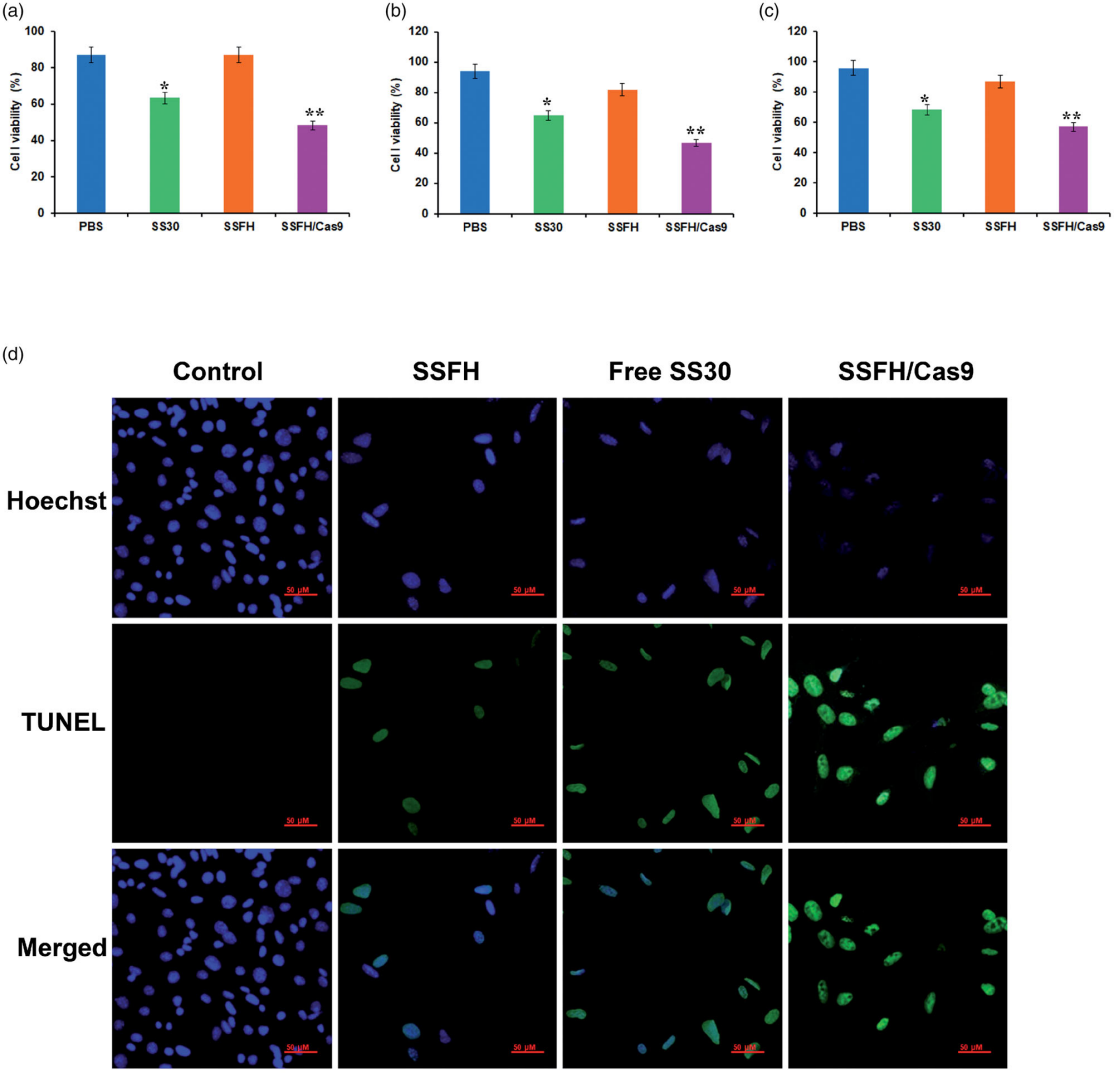
Anticancer ability evaluation of SSFH/Cas9 *in vitro*. (a–c) Proliferation inhibition ability of SSFH/Cas9 to CD123-positive cells. Different concentrations of SSFH/Cas9 was incubated with Molm-13 cells, cell proliferations were detected by MTS assay (a), CCK8 assay (b), and BrdU assay (c) (*p < .05, **p < .01). (d) Cell apoptosis assessment by TUNEL. Molm-13 cells were incubated with free SS30, SSFH, and SSFH/Cas9, respectively. Cell apoptosis were evaluated by TUNEL.

Since SS30 could inhibit proliferation of CD123-positive cells, to further evaluate the potential anti-proliferation effect of SSFH/Cas9 on CD123-positive cells, cell line Molm-13 was incubated with PBS, SS30, SSFH, SSFH/Cas9/sgRNA, or random library at 37 °C for 6 h, respectively. Then cells were washed by PBS and cultured for a further 48 h. To assess cell viability, both CCK8 and MTS assay were applied, while BrdU ELISA assay was used to detect cell proliferation. As presented in Figure 4(A) (MTS) and Figure 4(B) (CCK8), when compared with the PBS group, both SS30 and SSFH/Cas9/sgRNA significantly decreased cell viability of Molm-13 with statistical differences (SS30 group: *p* < .05; SSFH/Cas9 group: *p* < .01); However, SSFH did not obviously interfere cells viability of Molm-13 cells when compared with the PBS group. Furthermore, BrdU assay results are presented in Figure 4(C). Both SS30 and SSFH/Cas9/sgRNA significantly inhibited cells proliferation of Molm-13 cells with statistical differences (SS30 group: *p* < .05; SSFH/Cas9 group: *p* < .01), and SSFH, did not generate obvious inhibition ability due to restricted structures. Moreover, to further assess whether SSFH/Cas9 maintained CD123-positive cell apoptosis inducing ability of SS30, Molm-13 cells were treated with PBS, SS30, SSFH, and SSFH/Cas9/sgRNA for at 37 °C for 6 h. Cells were cultured for a further 48 h and subjected to TUNEL assay. As illustrated in Figure 4(D), when compared with the PBS group, both SS30 and SSFH/Cas9 significantly induced Molm-13 cell apoptosis; However, SSFH did not generate significant difference to Molm-13 of the PBS group. These results indicated that SSFH/Cas9 could effectively release SS30 and did not change the anti-cancer ability of SS30.

### SSFH/Cas9 could prolong retention time in vivo

**Figure 5.**
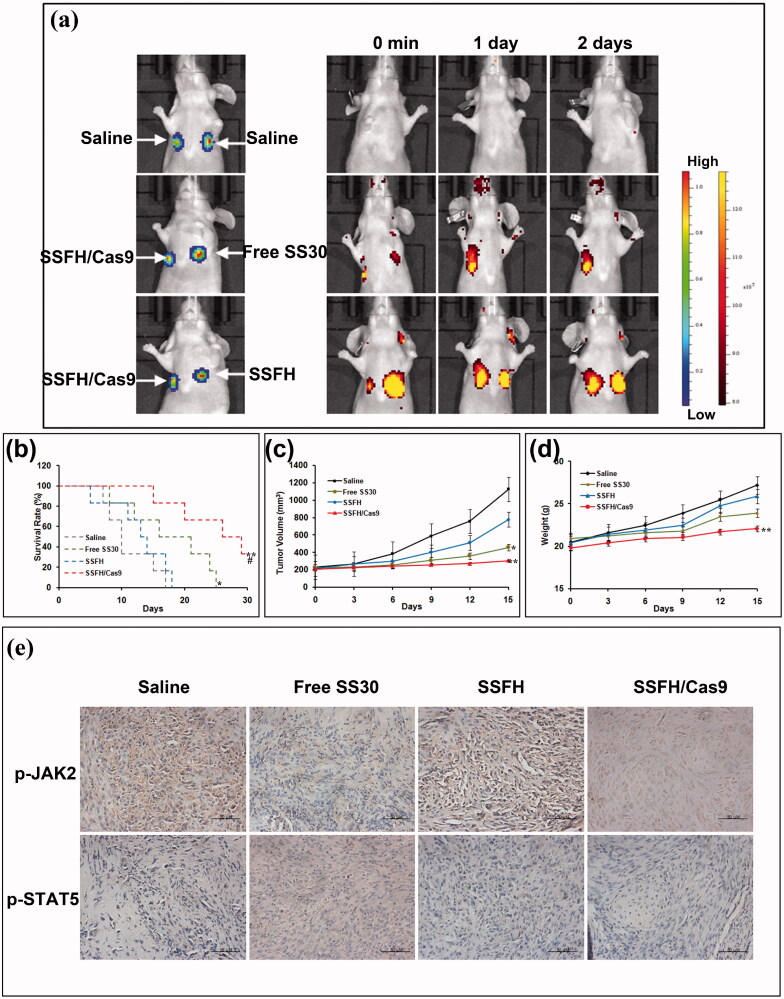
Anticancer ability evaluation of SSFH/Cas9 *in vivo*. (a) Retention of SSFH/Cas9 at the injection site. Tumor-bearing mice were subcutaneously injected with various samples tagged with Cy5.5 fluorescent probe. The retention of each sample at the injection site was observed using an IVIS® Spectrum CT. (b) Survival rate (%) of each group (* indicated *p* < .05 compared with the saline group; ** indicated *p* < .01 compared with the saline group; # indicated *p* < .05 compared with the free SS30 group). (c) Tumor volume of mice in each group (* indicated *p* < .05 compared with the saline group). (d) Weights of mice in each group (** indicated *p* < .01 compared with the saline group). (e) Histochemistry of mice tumor tissues. After initial treatment, mice were euthanized and tumor tissues were collected and expression of p-JAK2 and p-STAT5 were observed.

One purpose of designing SSFH was to prolong SS30 retention time *in vivo*, to assess whether SSFH could stay longer at the subcutaneous injection site of mice, SS30, SSFH, and SSFH/Cas9 were injected, and Cy5.5-tagged complementary probe was applied to visualize and be observed. As presented in Figure 5(A), SS30, SSFH, and SSFH/Cas9 differed in their retention at the subcutaneous injection site of mice. Both SSFH and SSFH/Cas9 were retained longer than the free SS30 aptamer in mice. Additionally, the SS30 group showed a rapid disappearance of the fluorescence signal within 1-day post-injection, whereas the SSFH and the SSFH/Cas9 group showed a prolonged fluorescence signal after administration. These suggested that a successful retention of SSFH *in vivo*, which may facilitate controllable administration, prolong internal circulation time, and further enhance drug efficacy and reduce drug consumption.

### SSFH/Cas9/sgRNA could inhibit CD123-positive tumors selectively in vivo

It should be evaluated that whether SSFH/Cas9 could inhibit CD123-positive cells *in vivo*. Therefore, Molm-13 cells were injected subcutaneously into the flank of BALB/c mice to generate the mouse xenograft tumor model. Fourteen days later, mice were divided into four groups: ① saline; ② SS30; ③ SSFH; ④ SSFH/Cas9/sgRNA. Treatments were administrated every 3 days. The mice survival rate is presented in Figure 5(B). When compared with the saline group, SSFH did not prolong survival time significantly since SS30 in SSFH could not be release without Cas9/sgRNA; Meanwhile, SS30 and SSFH/Cas9 could prolong survivals compared with the saline group due to anti-cancer ability of free SS30 (*p* < .05), and mice in the SSFH/Cas9 group survived longer than free SS30 (*p* < .05), due to a longer retention and function time. Tumor volume and body weight were also calculated to assess anti-cancer ability of different agents. As shown in Figure 5(C,D), body weights in the saline and the SSFH group increased rapidly due to a fast tumor growth, whereas the free SS30 and the SSFH/Cas9 group remained stably due to a much slower tumor growth. Interestingly, when compared with the free SS30 group, the SSFH/Cas9 group exhibited a smaller volume, this may due to a longer internal circulation and retention time for SS30 inhibition. Further, to assess whether SSFH/Cas9 inhibit by block IL-3/CD123 interaction and further interfere JAK2/STAT5 pathway, tumor tissues were collected and subjected to JAK2/STAT5 staining. As shown in Figure 5(E), the expression of p-JAK2 and p-STAT5 was significantly decreased in the SS30 and the SSFH/Cas9 group when compared with the saline and the SSFH group, indicating a successful inhibition of JAK2/STAT5 pathway.

### SSFH/Cas9/sgRNA system could rescue cytokine storm

**Figure 6.**
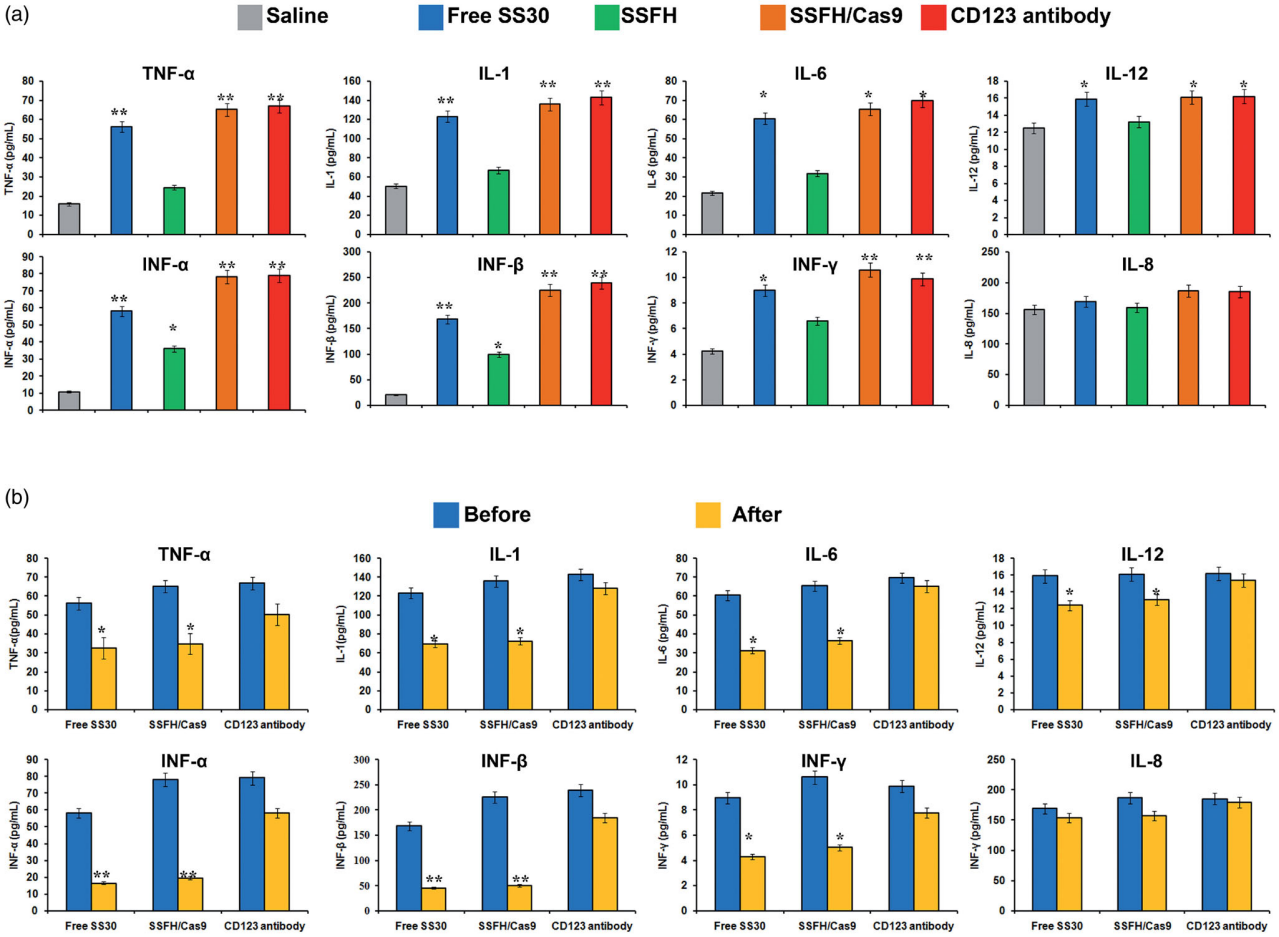
Serology assessment. (a) Blood samples of mouse xenograft tumor models were collected and subjected to detect inflammatory cytokines (TNF-α, IL-1, IL-6, IL-12, IFN-α, IFN-β, IFN-γ, and IL-8) (* indicated *p* < .05 compared with the saline group; ** indicated *p* < 0.01 compared with the saline group). (b) SSFH/Cas9 prevent development of cytokine storm. When the SSFH/Cas9 group and the CD123 antibody group exhibited an obvious difference compared with the saline group, agent injections should be ended. Mice in the saline group and the CD123 antibody group were injected with saline and mice in the SSFH/Cas9/sgRNA group were injected with the complementary sequence of SS30. Three days later, blood samples of mice were collected and subjected to detect inflammatory cytokines. (* indicated *p* < .05 compared with before SS30 complementary sequence injection; ** indicated *p* < .01 compared with before SS30 complementary sequence injection).

Since CD123 is also restrictedly expressed on normal cells such as monocyte cells which are mainly secreting cytokine, CD123 targeting agents may also damage these normal cells and induce cytokine storm, which is fatal in clinic. There was no effective method for cytokine storm rescue induced by CD123 targeted molecules. Therefore, to assess whether SSFH/Cas9/sgRNA system could exhibit a promising approach, the mouse xenograft tumor models were divided into three groups and injected with saline, excessive SSFH/Cas9/sgRNA or CD123 antibody to induce cytokine storm, respectively. Blood samples were collected and subjected to detect inflammatory cytokines (TNF-α, IL-1, IL-6, IL-12, IFN-α, IFN-β, IFN-γ, and IL-8). When the SSFH/Cas9/sgRNA group and the CD123 antibody group exhibited an obvious difference compared with the saline group, complementary sequence of SS30 were injected in the SSFH/Cas9/sgRNA group and inflammatory cytokines were evaluated 3 days later. The data are presented in Figure 6. It was obvious that after excessive injection, SSFH/Cas9/sgRNA and CD123 antibody both induced a higher concentration of cytokine when compared with the saline group (*p* < 0.05). Then, after the injection of complementary sequence of SS30 in the SSFH/Cas9/sgRNA group, the levels of cytokines decreased rapidly and were even to the saline group. However, although the CD123 antibody injection was ended, the cytokine levels were not declined to normal level. These data indicated a safer administration of SSFH/Cas9/sgRNA system to alleviate the side effects.

## Discussion

Based on our previous study, CD123 thioaptamer SS30 could inhibit CD123-positive AML cells via JAK2/STAT5 signaling pathway by blocking interaction between CD123 and IL-3. However, the molecular weight of SS30 was too small and it could be leaked from kidney rapidly, which may influence anti-cancer effects and short the interval of medication. Thus, the present study reports on a DNA hydrogel consisted with SS30 and could be controlled released by cleavage of Cas9 enzyme (Figure 1). This DNA hydrogel was termed as SSFH, and it has been validated that after Cas9/sgRNA incubation, SSFH could release a plenty of SS30 (Figures 2 and 3). The released SS30 could further inhibit CD123-positive cell proliferation targeted *in vitro*, prolong retention time and subsequently inhibit tumor growth *in vivo* (Figures 4–6).

Aptamers, a kind of novel targeting molecules, are single stranded DNA, RNA, or altered nucleic acids sequences generated from systematic evolution of ligands by exponential enrichment (SELEX) technology (Darmostuk et al., 2015). When compared with antibodies, aptamer could serve for various targets, including proteins, even cells, ions, etc. Aptamers have a unique three-dimensional spatial structure, which can bind to the target with high specific and affinity (Sun & Zu, 2015). Since aptamers were reported in 1992, so far, there have been plenty of aptamer explored. Aptamers have been used as diagnostic and therapeutic targeting ligands widely due to their obvious preponderance: a small molecular weight, stable structure, plasticity of chemical groups, fast blood clearance, and non-immunogenicity. Interestingly, some aptamers have the ability to regulate proteins functions and may inhibit their biological function of target proteins by binding to target the key domain of proteins (Kim et al., 2015; Chousterman et al., 2017; Khan et al., 2019). In our previous study, we have successfully developed a CD123 aptamer termed as SS30. SS30 has been validated that SS30 could bind with CD123 with high specificity and affinity. Additionally, SS30 could interfere the interaction between IL-3 and CD123, and further blocking JAK2/STAT5 signaling pathway, resulting in cell proliferation inhibition *in vitro* and *in vivo*. However, due to small size, SS30 could be leaked from kidney rapidly. How to prolong its circulatory time to enhance anti-cancer ability was a main issue. In this study, we first generated CD123 aptamer hydrogel (SS30/Cas9) which could be cut by Cas9/sgRNA. Currently, Cas9/sgRNA system has been used mainly in gene editing. Utilizing Cas9/sgRNA in controlled aptamer release may have advantages over previously reported aptamer delivery systems such as PLGA-conjugated aptamers. First, SSFH gel structure could resist to nuclease. SSFH contained a plenty of SS30 and most SS30 were folded inside and protected. Thus, when compared with other delivery systems, SSFH could prolong effects longer. Second, the specific cleavage of SSFH with Cas9 exhibited reducing off-target cleavage. Previous studies have explored cleavage by restriction enzyme. However, several reports have proven that restriction enzyme-mediated cleavage may encounter problems with nonspecific cleavage and inconsistencies in the tertiary structure of the released aptamers. This may due to shorter recognition region composed of 4–8 base pairs. In contrast, Cas9 needs 20-base pairs of double-stranded DNA to guide sgRNA. Therefore, Cas9/sgRNA offers an exquisitely specific cutting system and could provide higher precision (Chousterman et al., 2017). Additionally, the release of aptamers from SSFH hydrogel does not depend on the chemical reactions, which may reduce several chemical processes such as synthesis and purification. Moreover, the *in situ* biological release of aptamers by SSFH can eliminate such multi-step manufacturing processes for aptamer delivery when compared with other polymers.

It was notably that since CD123 was also restrictedly expressed on normal cells, resulting in side effects such as cytokines storm (Tong et al., 2020). Currently, cytokine storm syndrome (CSS) caused by CD123-targeted therapy is a major fatal factor during clinical treatment and hindered therapeutic effects severely (Chousterman et al., 2017). CSS is a serious and life-threatening disease characterized by systemic inflammation, ferritin, hemodynamics instability, and multiple organ failure (MOF). The common clinical features of CSS are persistent fever, splenomegaly, hepatomegaly with hepatic insufficiency, lymphadenopathy, coagulopathy, cytopenia, skin rash, and neurological symptoms (Kaur et al., 2020). The biomarker of CSS is an uncontrolled and dysfunctional immune response that involves the continuous activation and expansion of lymphocyte and macrophages, which secrete large amounts of cytokines, leading to cytokine storms. Currently, the treatments of CSS were mainly IFN-γ antibodies, IL-6 inhibitors, JAK inhibitors, IL-6 blocking therapy, and recombinant human IL-1Rα (Bayat et al., 2018). Aptamer, a kind of targeting molecules, exhibited obvious superiority when compared with antibodies. Aptamers are single oligonucleotides generated by *in vitro* selection mechanisms via the systematic evolution of ligand exponential enrichment (SELEX) process (Mirau et al., 2018). Aptamers can fold into three-dimensional structure to recognize and further bind with their targets with high specificities and affinities. Aptamers have attracted attention in targeted therapy because of their high binding affinity toward specific targets such as proteins, cells, small molecules, and even metal ions, antibodies for which are difficult to obtain Most importantly, the complementary sequences of aptamer could be utilized as antidote. Once there have generated obvious effects caused by aptamers, after injection of their complementary sequences, they could form double-strands immediately and destroy three-dimensional structure of aptamers (Krissanaprasit, 2021; Tabuchi, 2021; Zhao et al., 2021). Thus, in this study, to solve side effects caused by CD123 targeted molecules, we tried to assess whether complementary sequence of SS30 could alleviate cytokine storm caused by SSFH. As shown in Figure 6, both SSFH and CD123 antibody caused an obvious increasing of inflammatory cytokines. However, after injection of SS30 complementary sequence in the SSFH group, the levels of inflammatory cytokines decreased rapidly and were equal to the saline group, whereas the CD123 antibody group remained higher level until 3 days later. This indicated that aptamers showed more controllable with compared with antibodies. However, CD123 poly-aptamer hydrogel edited by Cas9/sgRNA still exited some disadvantages. The retention time *in vivo* could last a few days, but still needed to be improved. In addition, the possible immunogenicity of Cas9 which was used as a major component of this system should be addressed.

## Conclusion

Here in this study, we designed an RCA-based DNA hydrogel SSFH which could release CD123 aptamer SS30 by Cas9/sgRNA-mediated specific editing. It has been validated that DNA hydrogel SSFH could prolong retention time at tumor sites *in vivo*, which may facilitate anti-cancer ability of SS30. The use of Cas9/sgRNA as a precise editing system means that our system may be broadly applied to various aptamers and single-stranded DNA nucleotides. Although we just applied CD123 aptamer in this study, a successful retention and cleavage indicated that Cas9-edited controlled release of aptamers from RCA products could be utilized for other sustained release of aptamers from DNA hydrogels. Cas9/sgRNA-mediated specific editing of RCA-based DNA hydrogel may open a new approach for anti-cancer strategy and aptamer applications.
